# Type 1 Diabetes Mellitus Associated with Nivolumab after Second SARS-CoV-2 Vaccination, Japan

**DOI:** 10.3201/eid2807.220127

**Published:** 2022-07

**Authors:** Toshihiro Sato, Shinjiro Kodama, Keizo Kaneko, Junta Imai, Hideki Katagiri

**Affiliations:** Tohoku University Hospital, Sendai, Japan

**Keywords:** COVID-19, type 1 diabetes mellitus, nivolumab, severe acute respiratory syndrome coronavirus 2, SARS-CoV-2, coronaviruses, virus, coronavirus disease, respiratory infections, second SARS-CoV-2 vaccination, mRNA vaccine, immune checkpoint inhibitor, vaccines, zoonoses, Japan

## Abstract

Recently, along with increasing use of immune checkpoint inhibitors such as nivolumab, the incidence of immune-related adverse events, including type 1 diabetes mellitus, has become a serious problem. We report a patient who had immune checkpoint inhibitor‒associated type 1 diabetes mellitus that developed after a second mRNA-based SARS-CoV-2 vaccination.

In response to the COVID-19 pandemic, mRNA-based SARS-CoV-2 vaccination has spread rapidly worldwide. This vaccine has shown high efficacy for preventing infection and disease exacerbation. However, adverse immunologic effects, including myocarditis, have been reported (*1**,**2*). Thus, immune system disturbances induced by these vaccines are suspected.

Immune checkpoint inhibitors (ICIs), including nivolumab, target programmed cell death protein-1 and have been used to treat malignancies, including melanoma, nonsmall cell lung cancer, and renal cell carcinoma. However, immune-related adverse events, including type 1 diabetes mellitus (T1D), also develop after ICI therapies (*3*,*4*); this form of diabetes has been called immune checkpoint inhibitor‒induced diabetes mellitus (*5*). We report a patient who had checkpoint inhibitor‒induced diabetes mellitus develop after he received a second mRNA-based SARS-CoV-2 vaccination.

A 43-year-old man who had malignant melanoma (pT3bN1bM0 stage IIIC) received nivolumab treatment (480 mg 1× every 4 wks) 12 months before admission. Fasting plasma glucose level was 94 mg/dL and glycated hemoglobin (HbA1c) 5.6% at treatment initiation. Plasma glucose and HbA1c were tested every 4 weeks. His range of plasma glucose was 90–123 mg/dL and that of HbA1c was 5.4%–5.7% (Figure). Positron emission tomography–computed tomography showed no metastasis or recurrence of the tumor 1 month before admission.

**Figure:**
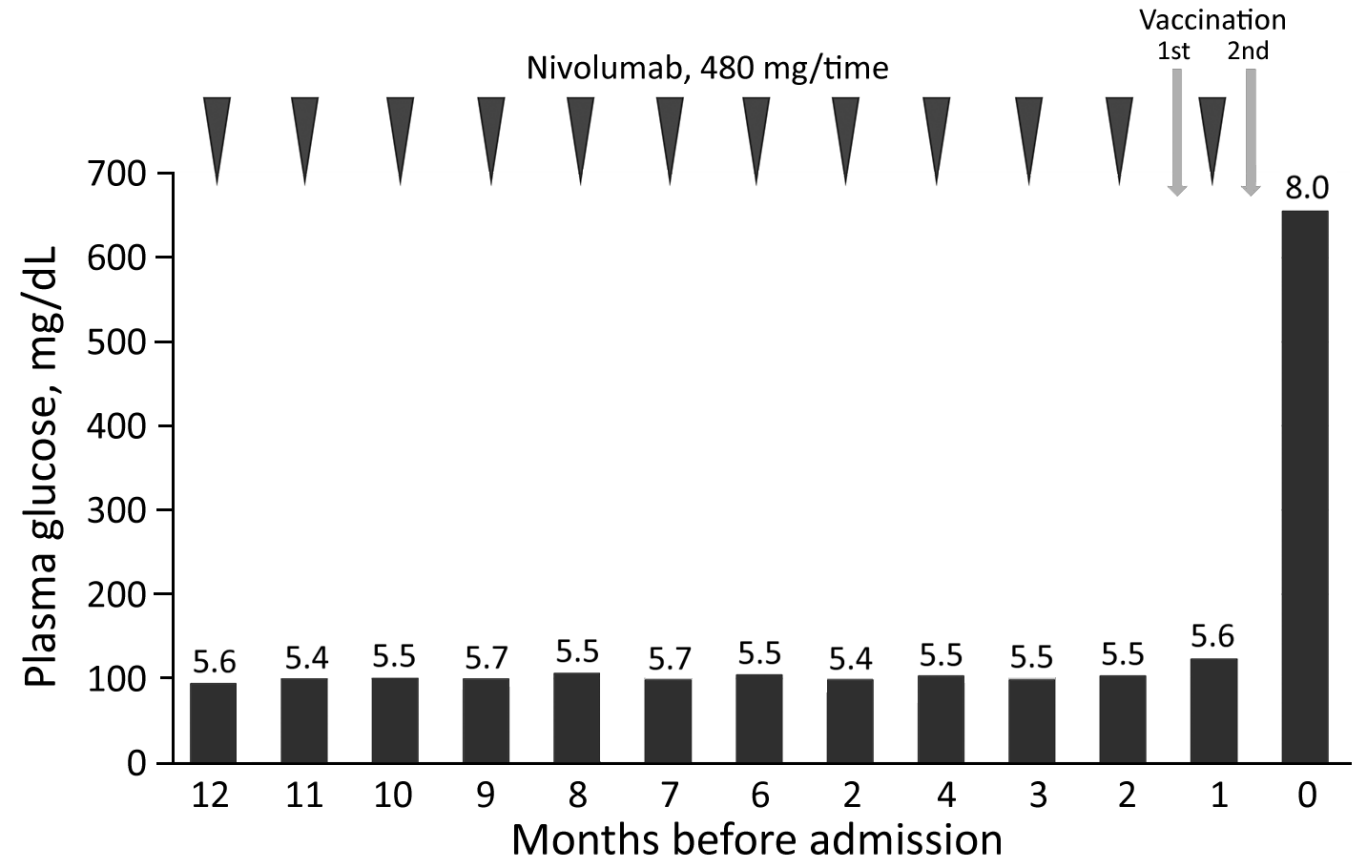
Clinical course after immune checkpoint inhibitors treatment initiation for type 1 diabetes mellitus associated with nivolumab after second SARS-CoV-2 vaccination, Japan. Numbers above bars are percentage glycated hemoglobin values.

The man received his first SARS-CoV-2 vaccination 35 days before admission. No apparent adverse reactions occurred, except for local pain. The last nivolumab dose was administered 21 days before admission and the second SARS-CoV-2 vaccination 14 days before admission. The next day, he had a slight fever (temperature 37°C), which soon subsided. Thirst, polydipsia, and polyuria appeared 2 days after the second vaccination. He started drinking 3 L of water/day, and his weight decreased by 5 kg over the next 12 days.

Twelve days after the second vaccination, his blood glucose level was 655 mg/dL and his HbA1c 8.0%. Levels of ketone bodies increased; 3-hydroxybutyric acid was 2,813 μmol/L and acetoacetate 1,936 μmol/L. He was urgently admitted to the hospital because of a diagnosis of ICI-associated T1D and marked ketosis.

Laboratory tests at admission showed severely impaired insulin secretion capacity; fasting C-peptide immunoreactivity (CPR) was 0.33 ng/mL, 24-hour urinary CPR 5.74 μg/day and 3.82 μg/day, and ΔCPR during the glucagon load test was 0.03 ng/mL (Table). Tests results for islet-specific autoantibodies against glutamic acid decarboxylase, insulinoma-associated antigen 2, and zinc transporter 8 were negative. Human leukocyte antigen typing identified no specific alleles, including DR4, known to be related to T1D (Table). Blood glucose decreased in response to continuous intravenous administration of insulin and saline. On the second day of hospitalization, we switched from intravenous to subcutaneous injection of insulin. The patient’s blood glucose level was ultimately controlled by intensive insulin therapy (degludec 9 U before dinner and lispro 24 U before breakfast, 5 U before lunch, and 15 U before dinner). Five months after discharge, the patient still requires multiple daily insulin injections for glycemic control.

**Table:** Laboratory test results for a patient who had type 1 diabetes mellitus associated with nivolumab after a second SARS-CoV-2 vaccination, Japan*

Laboratory test	Value
Venous blood gas analysis	
pH	7.36
pCO_2_, mm Hg	41.1
Bicarbonate, mmol/L	22.6
Anion gap, mmol/L	10.0
Biochemical	
Creatinine, mg/dL	0.84
Glomerular filtration rate, mL/min/1.73 m^2^	79.0
Amylase, U/L	59.0
Lipase, U/L	26.0
Diabetes-related tests	
Plasma glucose, mg/dL	665
HbA1c, %	8.0
Acetoacetate, μmol/L	1,936
3-hydroxybutyric acid, μmol/L	2,813
Fasting CPR, ng/mL	0.13
24-h CPR, μg/day†	5.74/3.82
GAD antibody, U/mL	<5.0
IA-2 antibody, U/mL	<0.4
ZnT8 antibody, U/mL	<10
Glucagon load test	
Fasting CPR, ng/mL	0.09
After 6 min CPR, ng/mL	0.12
Delta CPR, ng/mL	0.03
DNA typing	
HLA-DRB1*11:01-DQB1*03:01:01	NA
HLA-DRB1*13:02:01-DQB1*06:04:01	NA

*CPR, C-peptide immunoreactivity; GAD, glutamic acid decarboxylase; HbA1c, glycated hemoglobin; HLA, human leukocyte antigen; IA-2, insulinoma-associated antigen 2; NA, not applicable; ZnT8, zinc transporter 8. †The 24-h CPR was measured twice for this patient because values can fluctuate greatly for patients who have diabetes.

Recently, along with increasing use of ICIs, the incidence of immune-related adverse events, including T1D, has become a serious problem. Our patient had been receiving nivolumab for 1 year. During treatment, his blood glucose level was tested every 4 weeks, and no increases were detected. However, 2 days after the second mRNA vaccination, he had typical symptoms of severe hyperglycemia (i.e., thirst, polydipsia and polyuria, and subsequent weight loss). Fourteen days after the second vaccination, blood glucose level was markedly increased, and the patient had nearly depleted insulin secretion, but his HbA1c was <8.5%. Test results for islet-related autoantibodies were negative. Therefore, we gave the patient a diagnosis of the fulminant form of T1D (*6*), which was associated with ICI treatment.

Most patients with nivolumab-associated T1D reportedly have this complication develop within 7 months after ICI treatment initiation (*4*). However, for this patient, 12 months, an exceptionally long time, had elapsed when T1D manifested. Therefore, some other factor might have triggered onset of ICI-associated T1D.

SARS-CoV-2 mRNA vaccination reportedly alters immune conditions (*7*). The association between SARS-CoV-2 mRNA vaccines and myocarditis has recently received attention; myocarditis frequently occurred within 1 week, often just 2–4 days, after the second vaccination, mostly affecting young men (*1**,**2*). Clinical courses described were similar to that for our patient. Our patient had typical symptoms of severe hyperglycemia begin 2 days after the second SARS-CoV-2 vaccine dose. Thus, we speculate that the mRNA vaccine administered before manifestation of hyperglycemic symptoms might have triggered fulminant onset of T1D in this patient, who was at risk because of receiving ICI treatment.

Because of their high efficacy, mRNA vaccines should be applicable for inhibition of many diseases, not only viral infections but also malignancies. The clinical course of the patient we report suggests that caution should be exercised when administering mRNA vaccines, especially to persons at risk for autoimmune diseases, such as patients receiving ICI treatments, because T1D, particularly its fulminant onset, can be life-threatening if not promptly recognized and treated. However, we cannot rule out the possibility that T1D development in this patient was unrelated to the vaccination. Accumulation of similar observations would clarify the relationship between SARS-CoV-2 vaccination and development of T1D, especially that associated with ICI treatment.

## References

[1] Mevorach D, Anis E, Cedar N, Bromberg M, Haas EJ, Nadir E, et al. Myocarditis after BNT162b2 mRNA Vaccine against Covid-19 in Israel. N Engl J Med. 2021;385:2140–9. 10.1056/NEJMoa2109730 34614328 PMC8531987

[2] Witberg G, Barda N, Hoss S, Richter I, Wiessman M, Aviv Y, et al. Myocarditis after COVID-19 vaccination in a large health care organization. N Engl J Med. 2021;385:2132–9. 10.1056/NEJMoa2110737 34614329 PMC8531986

[3] Michot JM, Bigenwald C, Champiat S, Collins M, Carbonnel F, Postel-Vinay S, et al. Immune-related adverse events with immune checkpoint blockade: a comprehensive review. Eur J Cancer. 2016;54:139–48. 10.1016/j.ejca.2015.11.016 26765102

[4] Baden MY, Imagawa A, Abiru N, Awata T, Ikegami H, Uchigata Y, et al.; consultation of the Japan Diabetes Society Committee on Type 1 Diabetes Mellitus Research. Characteristics and clinical course of type 1 diabetes mellitus related to anti-programmed cell death-1 therapy. Diabetol Int. 2018;10:58–66. 10.1007/s13340-018-0362-2 30800564 PMC6357237

[5] Quandt Z, Young A, Anderson M. Immune checkpoint inhibitor diabetes mellitus: a novel form of autoimmune diabetes. Clin Exp Immunol. 2020;200:131–40. 10.1111/cei.13424 32027018 PMC7160652

[6] Imagawa A, Hanafusa T, Awata T, Ikegami H, Uchigata Y, Osawa H, et al. Report of the Committee of the Japan Diabetes Society on the research of fulminant and acute-onset type 1 diabetes mellitus: new diagnostic criteria of fulminant type 1 diabetes mellitus. J Diabetes Investig. 2012;3:536–9. 10.1111/jdi.12024 24843620 PMC4015434

[7] Sahin U, Muik A, Derhovanessian E, Vogler I, Kranz LM, Vormehr M, et al. COVID-19 vaccine BNT162b1 elicits human antibody and T_H_1 T cell responses. Nature. 2020;586:594–9. 10.1038/s41586-020-2814-7 32998157

